# Evolution of the Short Form of DNMT3A, DNMT3A2, Occurred in the Common Ancestor of Mammals

**DOI:** 10.1093/gbe/evac094

**Published:** 2022-06-23

**Authors:** Teruhito Ishihara, Danielle Hickford, Jane C Fenelon, Oliver W Griffith, Shunsuke Suzuki, Marilyn B Renfree

**Affiliations:** School of BioSciences, The University of Melbourne, Melbourne, Victoria 3010, Australia; School of BioSciences, The University of Melbourne, Melbourne, Victoria 3010, Australia; School of BioSciences, The University of Melbourne, Melbourne, Victoria 3010, Australia; Department of Biological Sciences, Macquarie University, Sydney, NSW 2109, Australia; Department of Agricultural and Life Sciences, Shinshu University, Nagano, Japan; School of BioSciences, The University of Melbourne, Melbourne, Victoria 3010, Australia

**Keywords:** marsupials, eutherians, monotremes, DNA methylation, genomic imprinting

## Abstract

Genomic imprinting is found in marsupial and eutherian mammals, but not in monotremes. While the primary regulator of genomic imprinting in eutherians is differential DNA methylation between parental alleles, conserved imprinted genes in marsupials tend to lack DNA methylation at their promoters. DNA methylation at eutherian imprinted genes is mainly catalyzed by a DNA methyltransferase (DNMT) enzyme, DNMT3A. There are two isoforms of eutherian DNMT3A: DNMT3A and DNMT3A2. DNMT3A2 is the primary isoform for establishing DNA methylation at eutherian imprinted genes and is essential for eutherian genomic imprinting. In this study, we investigated whether DNMT3A2 is also present in the two other mammalian lineages, marsupials and monotremes. We identified *DNMT3A2* in both marsupials and monotremes, although imprinting has not been identified in monotremes. By analyzing genomic sequences and transcriptome data across vertebrates, we concluded that the evolution of *DNMT3A2* occurred in the common ancestor of mammals. In addition, DNMT3A/3A2 gene and protein expression during gametogenesis showed distinct sexual dimorphisms in a marsupial, the tammar wallaby, and this pattern coincided with the sex-specific DNA methylation reprogramming in this species as it does in mice. Our results show that DNMT3A2 is present in all mammalian groups and suggests that the basic DNMT3A/3A2-based DNA methylation mechanism is conserved at least in therian mammals.

**Significance**

While eutherian genomic imprinting is mainly regulated by DNA methylation, marsupial imprinted genes tend to lack promoter DNA methylation. In this study, we asked whether this difference is caused by the absence of a primary enzyme, DNMT3A2. We confirmed that DNMT3A2 is present in all mammalian lineages. As the primary enzyme must have been evolved in the common ancestor of mammals, our result suggests that the difference in DNA methylation levels between marsupial and eutherian imprinting is caused by the associated proteins rather than the enzyme itself.

## Introduction

Genomic imprinting is an epigenetic process that causes differential expression of a subset of genes depending on their parental origin. Among vertebrates, genomic imprinting has been found in therian mammals, namely eutherians and marsupials, but there is no evidence for imprinting in monotremes or nonmammalian vertebrates (Pask et al. 2009; Renfree et al. 2009; Frésard et al. 2014; Griffith et al. 2016). Mammalian genomic imprinting is therefore thought to have evolved after the therian-monotreme divergence ∼184 million years ago (Cúneo et al. 2013). Genomic imprinting is known to regulate mammalian development and maternal behavior (Lefebvre et al. 1998; Reik and Walter 2001; Plagge et al. 2004; Keverne and Curley 2008; Renfree et al. 2009; Ferguson-Smith 2011), and disruption of imprinting causes many developmental defects such as placental abnormalities (Ono et al. 2006; Frost and Moore 2010; Piedrahita 2011). Although a number of hypotheses have been proposed for the roles of imprinted genes, questions remain as to how and why genomic imprinting evolved in therian mammals. As this phenomenon must have evolved in the common ancestor of therian mammals, characterizing similarities and differences in imprinting mechanisms between marsupials and eutherians, and comparing these to monotremes allow us to begin to characterize the ancestral mechanisms and to define how mammalian imprinting evolved.

The majority of the known-eutherian imprinted genes have certain regions marked by DNA methylation that differs in its methylation status between maternal and paternal alleles. Such regions are known as differentially methylated regions (DMRs; Bartolomei and Ferguson-Smith 2011). Differential DNA methylation status between the parental alleles is considered as the primary mechanism for genomic imprinting in eutherians. However, only three DMRs have so far been reported in marsupials (Weidman et al. 2006; Suzuki et al. 2007, 2018; Smits et al. 2008). Four conserved imprinted genes, *MEST*, *IGF2R*, *L3MBTL*, and *HTR2A*, which have a promoter DMR in eutherians (Stöger et al. 1993; Lefebvre et al. 1997; Riesewijk et al. 1997; Li et al. 2002, 2004; Joshi et al. 2016), lack a DMR on their promoter region in marsupials (Suzuki et al. 2005, 2018; Das et al. 2012). It is currently unknown how their imprinting status is established and maintained without a promoter DMR. In addition, marsupial imprinting is generally considered less stable than eutherian imprinting as it shows leaky expression from the silenced allele (Renfree et al. 2008). This indicates that eutherian mammals acquired additional molecular machineries for establishing more stable inactive marks like DNA methylation on promoters of genes such as *MEST* and *IGF2R* after the marsupial-eutherian split 166 million years ago.

In eutherians, DNA methylation is catalyzed by the DNA methyltransferase (DNMT) family of enzymes including DNMT3A, DNMT3B, and DNMT3L (Auclair and Weber 2012; Saitou et al. 2012; Sadakierska-Chudy et al. 2015). To ensure a sex-specific DNA methylation pattern is transmitted in gametes, the genome-wide DNA methylation status is reprogrammed in developing germ cells in both marsupial and eutherian mammals (Seisenberger et al. 2012; Monk 2015; Ishihara et al. 2019). In mice, this reprogramming is achieved by loss of parental DNA methylation marks followed by the resetting of DNA methylation by Dnmt3a, Dnmt3b and Dnmt3l in a sex-specific manner (Okano et al. 1999; Kaneda et al. 2004). In female mice, conditional KO of *Dnmt3a* reduces DNA methylation at the DMR of the conserved paternally expressed gene *Mest* (Kaneda et al. 2004), indicating that the DMR of this gene is established by the action of DNMT3A in eutherians. As *MEST* in marsupials is imprinted but does not have a DMR, comparisons of DNMT3A-based mechanisms between marsupials and eutherians could elucidate the differences in DMR-based imprinting between the two mammalian lineages.

There is a novel short isoform of DNMT3A, DNMT3A2, identified in eutherians (Chen et al. 2002; Sakai et al. 2004). Further characterization of DNMT3A2 demonstrated that it is a more effective isoform of DNMT3A at establishing DNA methylation on imprinted genes (Sakai et al. 2004; Nimura et al. 2006; Suetake et al. 2006; Ma et al. 2015; Thakur et al. 2016). Marsupials also have DNMT3A, DNMT3B and DNMT3L (Yokomine et al. 2006; Ishihara et al. 2019), but the presence of DNMT3A2 is unknown. It is therefore possible that the evolution of *DNMT3A2* occurred in the ancestor of eutherian mammals and caused the differences observed in the DNA methylation status of imprinted genes between eutherians and marsupials.

In this study, the evolution *DNMT3A2* was investigated through the identification of isoforms of *DNMT3A* across vertebrates focusing on the two other mammalian lineages, marsupials and monotremes. By analyzing transcriptome data of a marsupial (the koala: *Phascolarctos cinereus*), a monotreme (platypus: *Ornithorhynchus anatinus*), and non-mammals (chicken: *Gallus gallus*; the bearded dragon: *Pogona vitticeps*), we evaluated the presence of *DNMT3A2* across vertebrates. We further compared the gene and protein expression patterns of DNMT3As in the developing gonads of the marsupial tammar wallaby (*Macropus eugenii*) with the existing DNA methylation data for this species to determine conserved function. Here we report that the *DNMT3A2* occurred in all three mammalian groups so it must have evolved in the common ancestor of mammals and that the basic DNMT3A/3A2-based mechanism appears to be conserved at least in therian mammals.

## Results

### Tammar Wallaby has the Short Form of DNMT3A, DNMT3A2

**Fig. 1.**
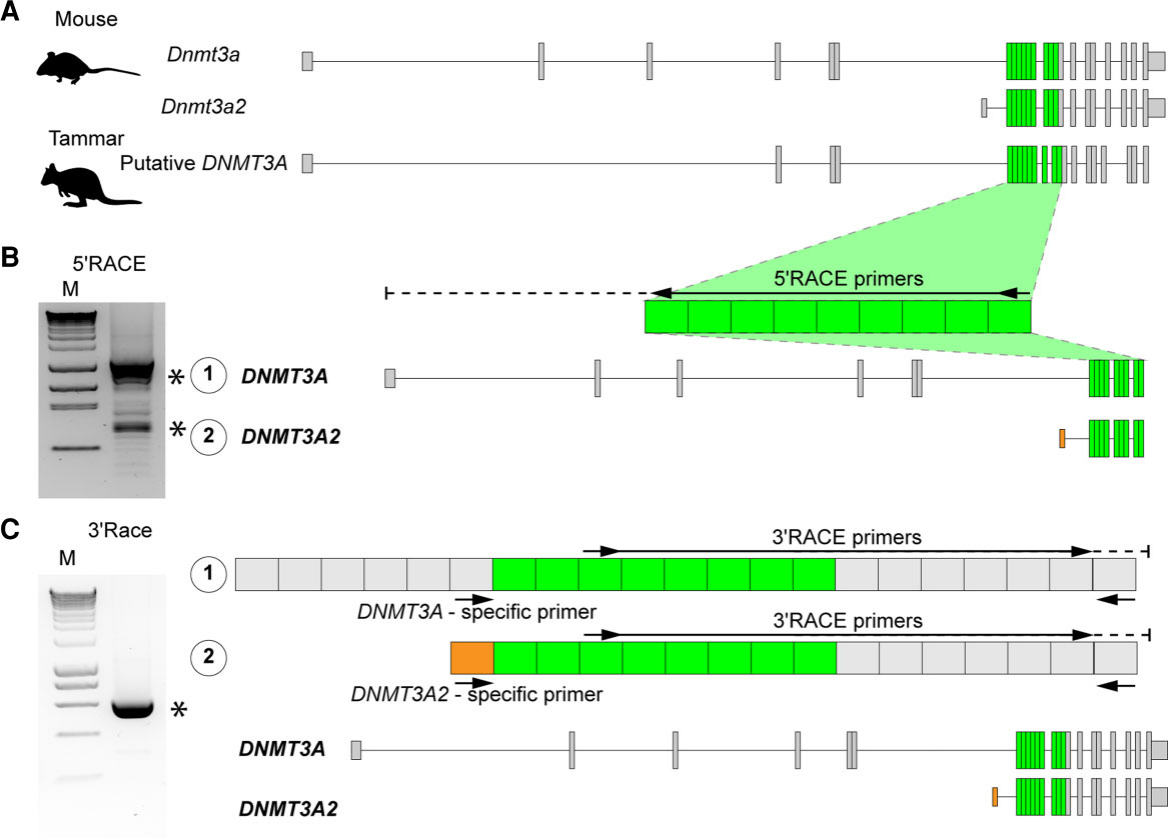
Identification of the tammar *DNMT3A2.* RACE experiments identified two isoforms of *DNMT3A* including *DNMT3A2* in the tammar. (*A*) Exon structures of mouse *Dnmt3a/3a2* and putative tammar *DNMT3A*. Conserved exons used for primer design were represented by green-colored boxes. (*B*) Identification of TSS of the tammar *DNMT3A* isoforms. 5′-RACE and nested RACE primers were represented by the black arrows. Asterisks represent RACE products coding partial tammar *DNMT3A* sequences. Boxes represent exons identified by sequencing of the RACE products. Orange-colored box shows *DNMT3A2*-specific exon. (*C*) Identification of the full length of the tammar *DNMT3A* and *DNMT3A2*. Standard PCR primers designed for characterizing each isoform and 3′-RACE primers were represented by the black arrows.

The 2,865 bp of tammar *DNMT3A* candidate sequence was identified by a blast search of the tammar genome database (Wallabase: https://wallabase.org). The comparison of the putative tammar *DNMT3A* with mouse *Dnmt3a/Dnmt3a2* identified highly conserved protein coding exons (fig. 1*A* and supplementary fig. S1). 5′-rapid amplification of cDNA end (RACE) reactions using a specific primer against the highly homologous region (fig. 1*A*) were then performed with adult tammar testis to characterize the marsupial *DNMT3A* isoforms. The 5′-RACE products showed two distinct bands (fig. 1*B*), indicating that there were two isoforms. After cloning and sequencing of each RACE product, two transcript variants of *DNMT3A* were identified. The first exon of the shorter isoform was located between exon 6 and exon 7 of the longer isoform. Based on the exon structure, we renamed the shorter isoform as *DNMT3A2* (fig. 1*C*). Combining specific primers against the *DNMT3A2* and 3′-RACE reactions confirmed that the tammar *DNMT3A2* is also a protein coding transcript (fig. 1*C*).

### Platypus also has DNMT3A2 and a Unique Isoform of DNMT3A, DNMT3AOa

**Fig. 2.**
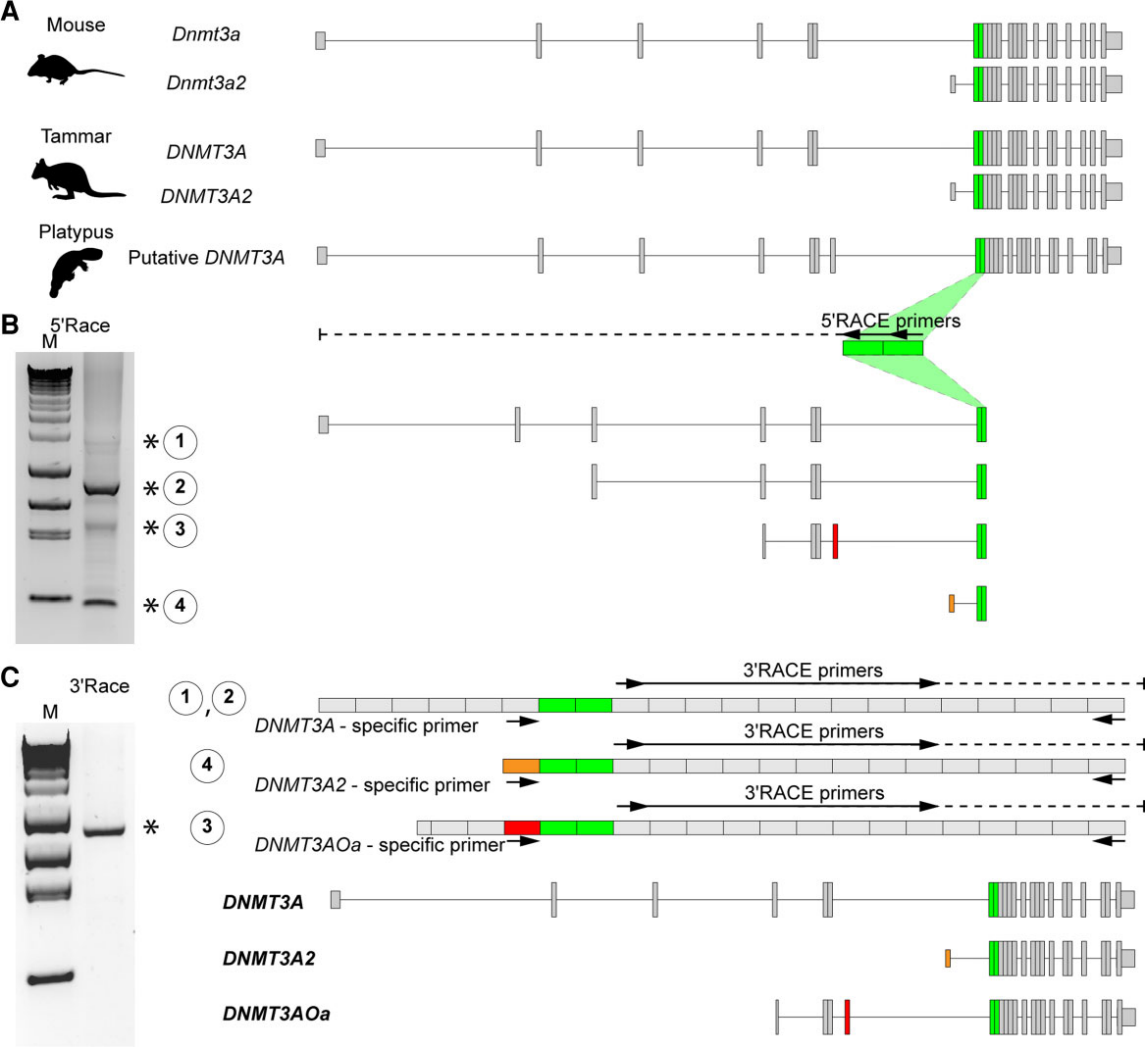
Identification of platypus *DNMT3A2.* RACE experiments identified four isoforms of *DNMT3A* including *DNMT3A2* in platypus. (*A*) Exon structures of mouse *Dnmt3a/3a2*, tammar *DNMT3A/3A2* and putative platypus *DNMT3A*. Conserved exons used for primer design were represented by green-colored boxes. (*B*) Identification of TSS of platypus *DNMT3A* isoforms. 5′-RACE and nested RACE primers were represented by the black arrows. Asterisks represent race products coding partial platypus *DNMT3A* sequences. Boxes represent exons identified by sequencing of the RACE products. Orange- and red-colored boxes show *DNMT3A2*-specific and *DNMT3AOa*-specific exons, respectively (*C*) Identification of the full length of the platypus *DNMT3As*. Standard PCR primers designed for characterizing each isoform and 3′-RACE primers were represented by the black arrows.

The 2,865 bp of platypus *DNMT3A* candidate sequence was identified by a blast search of the new platypus genome (mOrnAna1.p.v4). 5′-RACE reactions were then performed using adult platypus testis to identify monotreme *DNMT3A* isoforms (fig. 2*A*). The 5′-RACE products showed four amplicons, indicating that there were four isoforms. After sequencing each RACE product, four transcript variants of *DNMT3A* were identified. Isoform 4 encoded the shortest form of *DNMT3A* and its first exon was located between exon 6 and exon 7 of the longest form. Based on the exon structure, we renamed the shortest isoform as *DNMT3A2* (fig. 2*B*). Using specific primers against the *DNMT3A2* and *isoform3*, 3′-RACE reactions confirmed that the platypus *DNMT3A2* and *isoform3* were protein coding transcripts (fig. 2*C*). *Isoform 3* contained a unique exon and encoded a unique protein. A BLAST search using NCBI BLAST did not reveal any similar sequences with this unique exon. Thus, we named *isoform 3* as a novel isoform, *DNMT3A O. anatinus* specific isoform, *DNMT3AOa* (fig. 2*C*).

### The Transcription Start Site of DNMT3A2 is Conserved Across Mammals

**Fig. 3.**
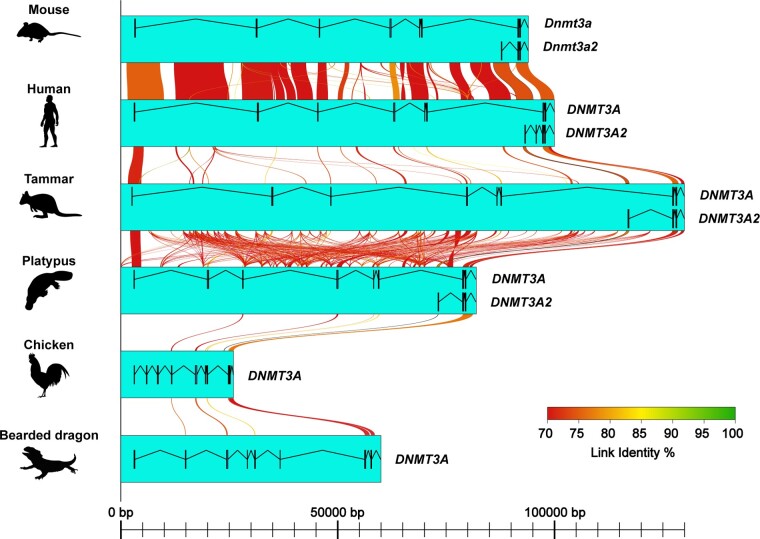
TSSs of *DNMT3A2* are conserved across mammals. Multiple alignments confirmed presence of highly conserved region at the TSS of *DNMT3A2* in mammals. Genomic sequences of mammals (mouse, human, tammar, and platypus), chicken, and the central-bearded dragon were aligned, and their sequence homologies were visualized with AliTV program. The transcription start sites of *DNMT3A2* were conserved across mammals. The first exon of platypus *DNMT3A2* had a homology with chicken genome, although it was <62 bp. Exon structures of *DNMT3A* and *DNMT3A2* are shown as black box connected with black lines in the aqua boxes.

To identify conserved cis-regulatory elements at the transcription start site (TSS) of *DNMT3A2* in mammals, genomic alignment was performed and visualized with AliTV program (Ankenbrand et al. 2017). The TSS of *DNMT3A2* was highly conserved across mammals (fig. 3). Note that the TSS of platypus *DNMT3A2* also has high homology with a region in the chicken *DNMT3A* locus. However, the range of the region is <62 bp and the sequence does not encode the first exon of mammalian *DNMT3A2*. The TSS of mammalian *DNMT3A2* showed no homology with the *DNMT3A* locus of the bearded dragon lizard (*P. vitticeps*).

**Fig. 4.**
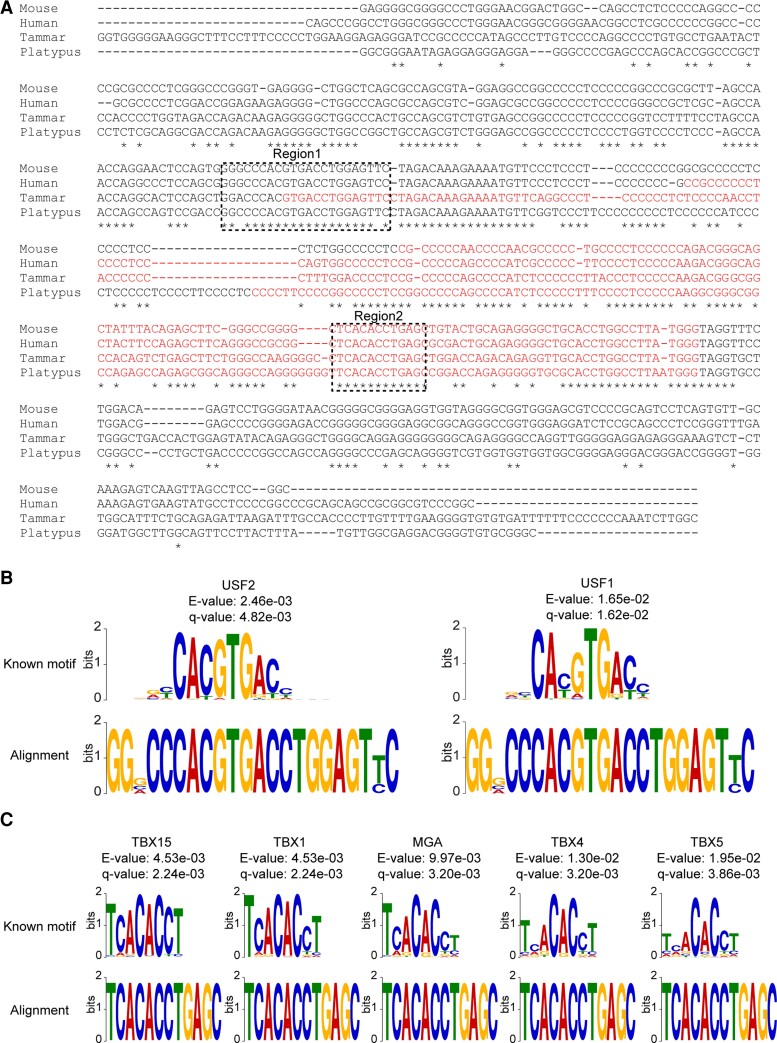
Common cis-acting elements around the TSS of *DNMT3A2* across mammals. Multiple alignments of the TSS of mammalian *DNMT3A2* identified common cis-acting elements. (*A*) Multiple alignments of the highly conserved region around the TSS of *DNMT3A2* in mammals. The first exon of *DNMT3A2* is shown in red-colored letters. There are two regions (regions 1 and 2) which had significant matches (*E*-value <0.05 and *q*-value <0.05) based on TOMTOM program in MEME suite. (*B*) Motif structure of the region 1. The region contained upstream transcription factor 2 (USF2) and USF1 binding motifs. (*C*) Motif structure of the region 2. The region contained T-box transcription factors (TBX15, TBX1, TBX4, TBX5) binding site and Max dimerization protein (MGA) binding site.

To identify common cis motifs around the TSS of *DNMT3A2*, the highly homologous region identified across mammals was subjected to multiple alignments by clustalW. As a result, highly conserved sequences around the TSS of *DNMT3A2* across mammals were identified (fig. 4*A*; asterisks). Regions containing five or more highly homologous sequences were compared with known transcription factor binding motifs using the TOMTOM program in the MEME suite. From this analysis, two common regions were found that had significant matches with *E*-values <0.05 and *q*-values <0.05 (fig. 4*A*; dotted boxes). Region 1 contained upstream transcription factor 2 (USF2) and USF1 binding motifs (fig. 4*B*), and region 2 contained a T-box transcription factors (TBX15, TBX1, TBX4, TBX5) binding site and a Max dimerization protein (MGA) binding site (fig. 4*C*). Region 2 was downstream of the TSS and within the first exon of mammalian *DNMT3A2*.

### DNMT3A2 is a Mammal Specific Isoform of DNMT3A

**Fig. 5.**
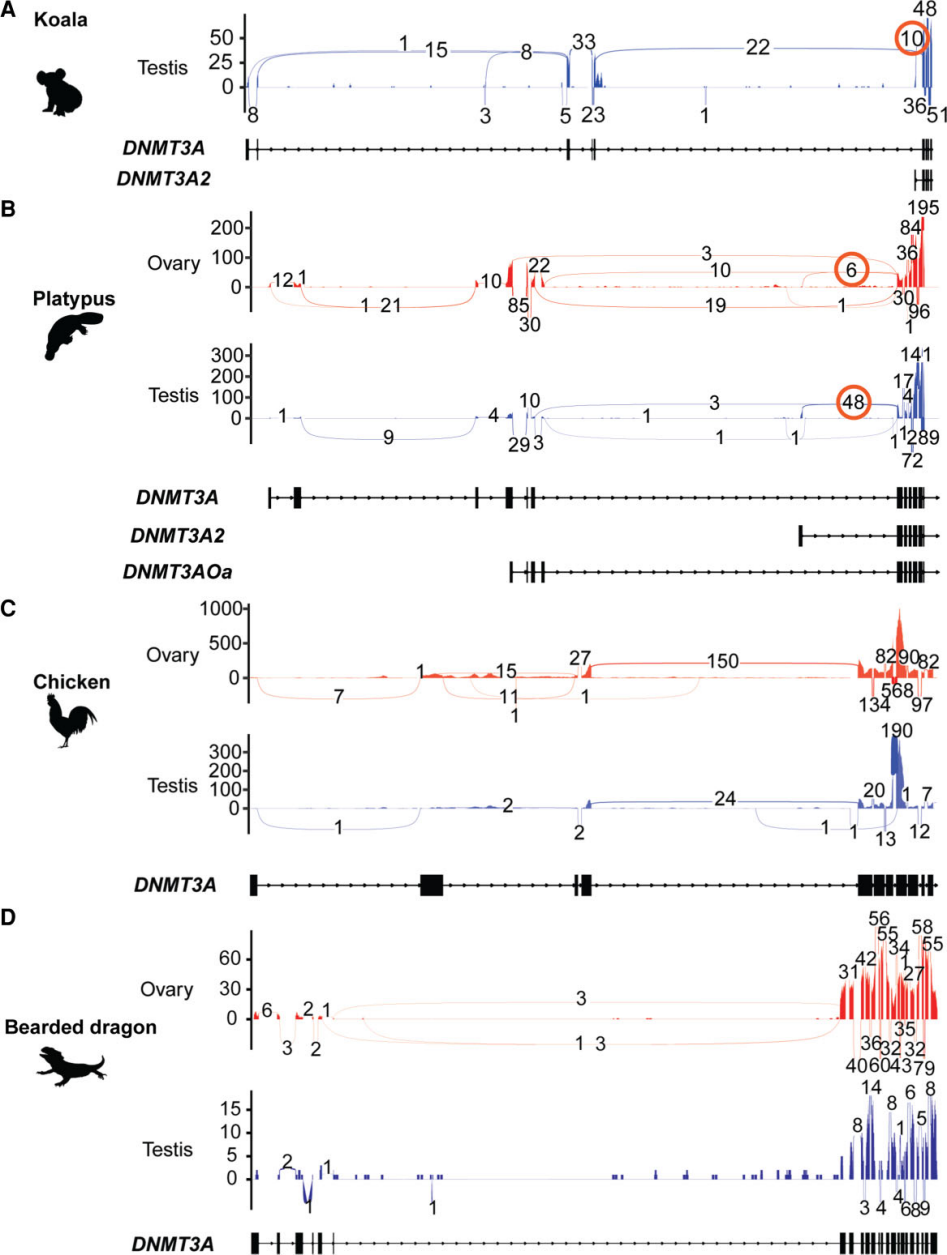
Visualizations of splicing junctions of *DNMT3A* confirmed *DNMT3A2* in koala and platypus but not in other vertebrates. Visualizations of splicing junctions of vertebrate *DNMT3A* identified *DNMT3A2* in koala and platypus but not chicken and bearded dragon. Published gonadal transcriptome data sets derived from koala, platypus, chicken, and a dragon lizard were mapped against their genomic sequences and splicing junctions of *DNMT3A* were visualized by sashimi plot. Splicing junctions were shown as lines connecting two exons and numbers of junction reads were shown on the junction lines. Exon structures of *DNMT3A* were shown as black boxes under the sashimi plot. (*A*) Koala. In testis, koala transcriptome data analysis successfully detected the short form, *DNMT3A2* (orange circle). (*B*) Platypus. In both sexes, platypus transcriptome data analysis successfully detected the short form, *DNMT3A2* (orange circle). (*C*) Chicken. Chicken transcriptome data analysis could not identify *DNMT3A2* in gonads. (*D*) The central-bearded dragon, *Pogona vitticeps*. Transcriptome data analysis did not identify *DNMT3A2* in gonads.

Published transcriptome data sets of gonads derived from chicken and the bearded dragon were analyzed to identify potential isoforms by visualizing splicing junctions. Koala and platypus transcriptome data sets were also analyzed to evaluate the isoform detection method based on visualizing splicing junctions. Visualizing splicing junctions successfully identified the short form *DNMT3A2* from both the koala and platypus transcriptome data (fig. 5*A* and *B*). In the platypus, the presence of *DNMT3AOa* was also confirmed in both male and female gonadal transcriptome data by detecting reads mapped to the unique exon of the isoform (fig. 5*B*). However, chicken and bearded dragon data sets clearly lacked the short form (fig. 5*C* and *D*).

### Expression Patterns of DNMT3A/A2 During Gametogenesis are Conserved Across Therian Mammals

**Fig. 6.**
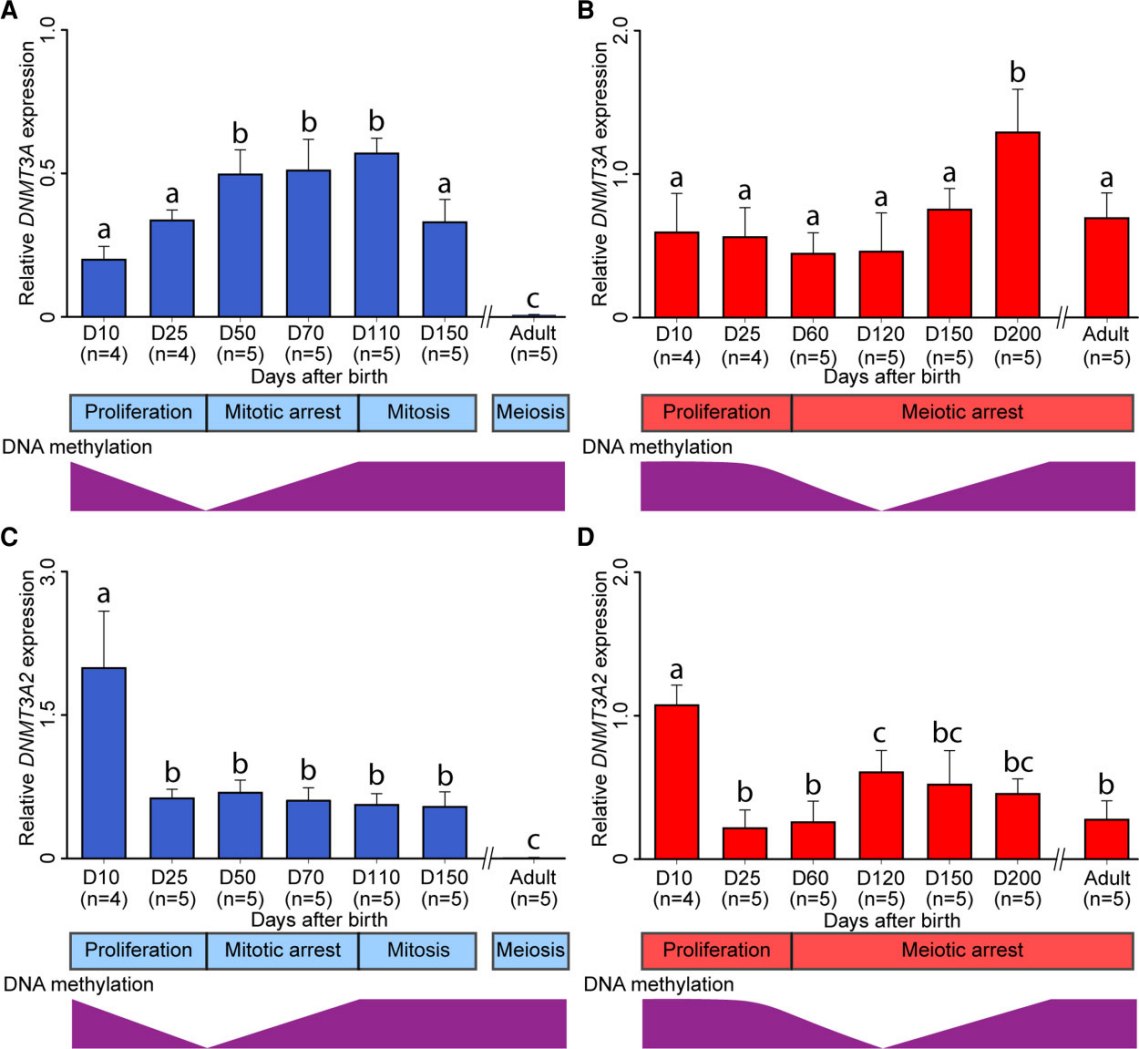
Expression of *DNMT3A* and *DNMT3A2* during tammar gametogenesis. *DNMT3A*/*3A2* gene expression during gametogenesis was consistent with DNA methylation reprogramming in the tammar. (*A*) *DNMT3A* expression during spermatogenesis. (*B*) *DNMT3A* expression during oogenesis. (*C*) *DNMT3A2* expression during spermatogenesis. (*D*) *DNMT3A2* expression during oogenesis. Relative gene expression against two housekeeping genes, *HMBS* and *TBP*, was shown as bar graph. Error bar represents standard error of the mean (SEM). Bars labelled with the same letters represent values that do not differ significantly by Tukey–Kramer’s multiple comparison test at *P* < 0.05. Sample numbers of each stage are represented in parentheses. The corresponding developmental stages of male and female tammar germ cells based on pervious publications (Ullmann et al. 1997; Ishihara et al. 2021) are shown at the bottom. DNA methylation data were based on a previous study (Ishihara et al. 2019).

Real-time reverse transcription-polymerase chain reaction (RT-PCR) experiments using developing gonads from the tammar were performed to confirm the correlation between *DNMT3A/A2* expression and the existing data on tammar DNA methylation reprogramming during gametogenesis. During oogenesis, tammar *DNMT3A* expression was upregulated at day 200 pp, after folliculogenesis begins (Mattiske et al. 2002;fig. 6*A*). During spermatogenesis, tammar *DNMT3A* expression was significantly upregulated during mitotic arrest when most of the DNA methylation is re-established (Ishihara et al. 2019) (fig. 6*B*). In both sexes, tammar *DNMT3A2* showed significantly higher expression at day 10 pp compared with the other ages examined in this study (*P* < 0.05). After day 25 pp, tammar *DNMT3A2* expression was downregulated in both sexes (fig. 6*C* and *D*). In females, tammar *DNMT3A2* expression was significantly upregulated (*P* < 0.05) at day 120 pp when DNA remethylation begins (Ishihara et al. 2019) and then gradually downregulated as development proceeds (fig. 6*C*). In contrast to females, male tammar *DNMT3A2* expression was constant during prespermatogenesis and significantly decreased (*P* < 0.05) in the adult testis (fig. 6*D*).

### DNMT3A/3A2 Protein became Nuclear at the Time of DNA Remethylation in Both Sexes

**Fig. 7.**
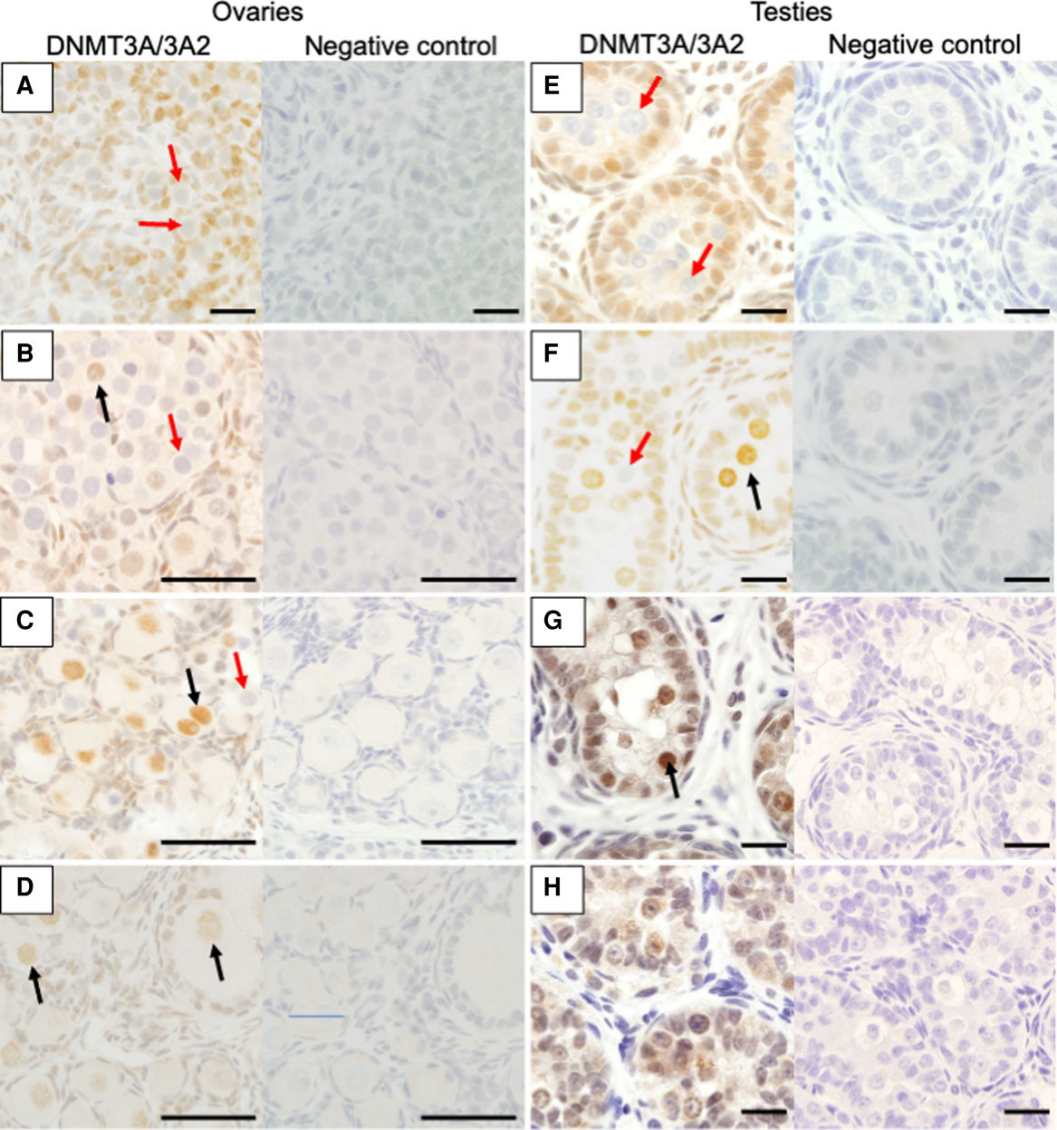
DNMT3A/3A2 protein localization during tammar gametogenesis. Immunohistochemistry of DNMT3A/3A2 during oogenesis and spermatogenesis revealed sex-specific DNMT3A/3A2 upregulation in developing germ cells. (*A*) Ovary at day 16 pp. (*B*) Ovary at day 60 pp. (*C*) Ovary at day 110 pp. (*D*) Ovary at day 192 pp. (*E*) Testis at day 26 pp. (*F*) Testis at day 60 pp. (*G*) Testis at day 120 pp. (*H*) Testis at day 300 pp. Black and red arrows represent germ cell with DNMT3A/3A2 staining and DNMT3A/3A2 negative germ cells, respectively. Brown color: positive staining. Scale bars: 20 μm.

To further confirm the correlation between DNA methylation reprogramming and DNMT3A/3A2 expression, immunohistochemistry (IHC) was performed. Based on our western blotting analysis, the antibody used in this study could detect both DNMT3A and DNMT3A2 (supplementary fig. S2). In developing gonads, tammar DNMT3A/3A2 protein localization was always nuclear (fig. 7). Within ovaries, at day 16 pp, DNMT3A/3A2 was restricted to somatic cells (fig. 7*A*). By day 60 pp, when early folliculogenesis has commenced, several oocytes within primordial follicles adjacent to the cortex were DNMT3A/3A2-positive (fig. 7*B*). At day 120 pp, when DNA remethylation begins (Ishihara et al. 2019), some oocytes contained nuclear DNMT3A/3A2 but others were negative, seemingly with no respect to oocyte size or location (fig. 7*C*). At day 230 pp, some smaller oocytes within primary follicles had nuclear DNMT3A, as did most larger oocytes within secondary and tertiary follicles (fig. 7*D*).

At day 26 pp, the majority of germ cells were DNMT3A/3A2 negative (fig. 7*E*). At day 60 pp when DNA remethylation has commenced (Ishihara et al. 2019), it appeared there were more DNMT3A/3A2-positive cells compared with day 26 pp (fig. 7*F*). Day 110 pp testes contained similar numbers of DNMT3A/3A2-positive cells to day 60 pp (fig. 7*G*). However, in day 300 pp testes, DNMT3A/3A2-positive germ cells were not evident compared with day 110 pp (fig. 7*H*).

## Discussion

**Fig. 8.**
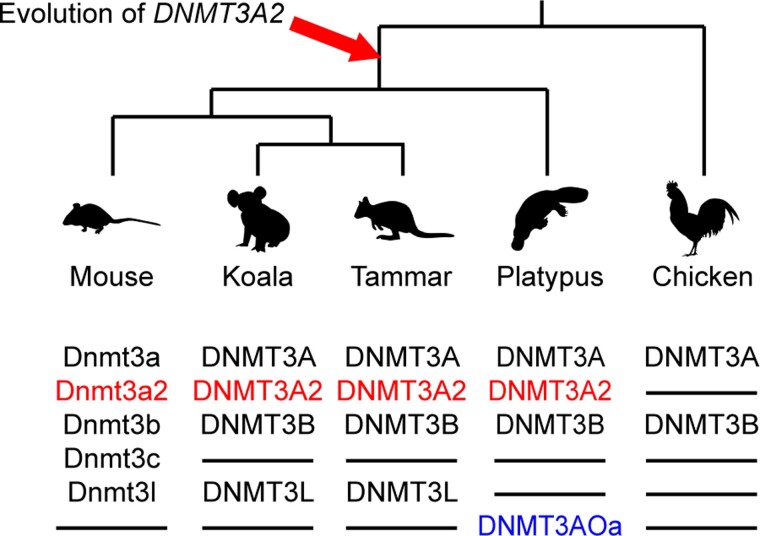
Evolution of *DNMT3A2* occurred in the common ancestor of mammals. Phylogenetic tree and DNMT3s. DNMT3 family member proteins of each animal are listed under the silhouette of each animal. The important isoform of DNMT3A, DNMT3A2 has evolved in the common ancestor of mammals (red letters). The platypus lacks a cofactor of DNMT3A, DNMT3L, but the platypus has the platypus-specific isoform of DNMT3A, DNMT3AOa (blue letters).

The short form of *DNMT3A*, *DNMT3A2* is critical in establishing DMR-based imprinting in eutherians. As most marsupial imprinted genes so far identified do not have DMRs, we predicted that the evolution of DNMT3A2 occurred after the eutherian-marsupial split 166 million years ago. However, *DNMT3A2* was present not only in marsupials but also in monotremes, in which genomic imprinting has not been identified. No *DNMT3A2* was identified in the chicken or the bearded dragon, but the TSS of *DNMT3A2* was conserved across all mammals, suggesting that the acquisition of the new promoter for *DNMT3A2* expression occurred in the common ancestor of mammals before the monotreme-therian split over 184 million years ago (Cúneo et al. 2013; fig. 8).

The highly homologous region around the TSS of *DNMT3A2* across mammals contained a number of consensus binding motifs. The putative USF1/2 binding sites were located in the TSS of the tammar *DNMT3A2* and upstream of the TSS of *DNMT3A2* in the other mammals examined in this study. USFs generally bind to genomic regions close to TSS of target genes and regulate their expression (Rada-Iglesias et al. 2008). For example, USF1 binds to the first exon of the *N4bp2l1* gene and regulate its expression in mice (Watanabe et al. 2019). This suggests that USF1/2 binding may contribute to the expression of *DNMT3A2* in mammals. Moreover, we found a putative binding site of T-box transcription factors such as TBX15 and TBX5. The putative TBX factor binding site was downstream of the TSS of *DNMT3A2*. However, Tbx5 binds to a motif located downstream of the TSS of *Fgf10* and controls its expression in developing mouse limbs (Delgado et al. 2021). Thus, T-box transcription factors regulate target gene expression even when they bind to binding sites downstream of the TSS. These data suggest that these T-box transcription factors may regulate *DNMT3A2* expression in mammals as seen in mouse *Fgf10*. TBX15 is known to be required for skeletal development of the limb, vertebral column, and head (Singh et al. 2005). In mice, Dnmt3a/3a2 deficiency shows defects in osteoclast differentiation (Nishikawa et al. 2015). The other T-box transcription factor, TBX5, is involved in cardiovascular development (Steimle and Moskowitz 2017), and inactivating DNMT3A/3A2 contributes to cardiovascular disease (Sano et al. 2018). Therefore, the short isoform, DNMT3A2, in addition to establishing DNA methylation-based imprinting in germ cells, may have a functional role in mammalian bone and cardiovascular development. Further characterization of the molecular mechanisms that regulate *DNMT3A2* expression could elucidate possible links between *DNMT3A2*, T-box transcription factors and mammalian development.

A cofactor of DNMT3A, DNMT3L is essential for adding DNA methylation on selected gene loci, including imprinted genes in eutherians (Bourc’his et al. 2001; Hata et al. 2002; la Salle et al. 2007; Glass et al. 2009). Across mammals, *DNMT3L* is conserved in both marsupials and eutherians but not in monotremes (Yokomine et al. 2006; Renfree et al. 2009). These data suggest that monotremes lack an ability to recruit DNA methylation on selected gene loci to establish genomic imprinting. However, in this study, the novel platypus-specific isoform DNMT3AOa was identified. Although the exact function of DNMT3AOa is currently unknown, this novel isoform may increase the capacity for the platypus to add DNA methylation onto their genome. The capacity of DNMT3AOa to add DNA methylation on platypus genome needs to be determined.

In mice, *Dnmt3a/3a2* expression shows distinct sexual dimorphisms during gametogenesis and correlates with DNA methylation reprogramming in both sexes (la Salle et al. 2004; Lucifero et al. 2007). Our analysis showed that patterns of tammar *DNMT3A* and *DNMT3A2* expression were also different between the sexes during gametogenesis and correlated with the known DNA methylation patterns across gametogenesis in the tammar. Although the previous mouse studies did not separate *Dnmt3a2* expression from *Dnmt3a* expression in developing germ cells (la Salle et al. 2004; Lucifero et al. 2007), the combined expression of mouse *Dnmt3a/Dnmt3a2* and tammar *DNMT3A/DNMT3A2* have similar patterns during gametogenesis. Our IHC analysis further confirmed that DNMT3A/3A2 localization also showed sex-specific differences consistent with DNA methylation reprogramming in this species. These data suggest that the sex-specific global DNA methylation reprogramming in therian mammals is mediated by a conserved DNMT3A/DNMT3A2-based mechanism. Although we could not confirm *DNMT3A/3A2* expression in developing platypus gonads due to limited access to samples, it would be interesting to determine if this pattern is conserved across all mammals.

As well as DNMT3A/3A2 expression, the expression pattern of its co-factor *DNMT3L* in the tammar is also similar to mouse *Dnmt3l* (Ishihara et al. 2019). Therefore, based on this study and previous studies (Yokomine et al. 2006; Renfree et al. 2009; Ishihara et al. 2019), we confirmed that marsupials have all the known molecular machineries, DNMT3A/3A2 and DNMT3L, required for catalyzing DNA methylation on imprinted genes. Despite this, some marsupial imprinted genes lack promoter DNA methylation. For example, in the tammar, the conserved imprinted gene *MEST* lacks a DMR on its promoter (Suzuki et al. 2005, 2018; Das et al. 2012). However, in mice, *Mest* DMR establishment in oocytes occurs in the later stages of oogenesis (Lucifero et al. 2004) and during tammar oogenesis, late upregulation of *DNMT3A* relative to *DNMT3A2* was observed. As the combined expression of mouse *Dnmt3a/Dnmt3a2* and tammar *DNMT3A/DNMT3A2* has similar patterns during oogenesis, this suggests that the lack of DNA methylation on the promoter of marsupial *MEST* might be caused by differences in DNMT3A and/or its associated proteins between marsupials and eutherians. However, confirmation of this requires examination of the specific expression pattern of Dnmt3a relative to Dnmt3a2 in the mouse during oogenesis.

This study confirms that the overall mechanism and expression pattern of DNMT3A/DNMT3A2 is conserved among therian mammals. However, given this conservation, it remains unclear as to why some marsupial imprinted genes lack DMRs on their promoters. DNMT3A2 was also present in monotremes. In addition, a novel DNMT3A isoform was identified in monotremes which may contribute to establish the global DNA methylation levels. Further examination of monotremes could provide important insights into evolution of mammalian genomic imprinting.

## Materials and Methods

### Animals

Tammar wallaby (*M. eugenii*) samples of Kangaroo Island, South Australia origin, were collected from captive animals from our colony maintained by the University of Melbourne. Gonads were snap-frozen immediately after dissection. Platypus (*O. anatinus*) tissues were collected from wild-caught animals under permits from NSW Parks and Wildlife and ethically approved by the University of Melbourne Animal Ethics committees. All animal handling and husbandry were in accordance with the National Health and Medical Research Council of Australia (2013) guidelines.

### Gonadal RNA Extraction and cDNA Synthesis

Snap-frozen gonads were used for RNA extraction using the GenElute Mammalian total RNA Miniprep Kit (Sigma-Aldrich, St Louis, MO, USA) following the manufacturer’s instructions. The extracted RNA was treated with the DNA-free DNase treatment and removal kit (Thermo Fisher Scientific, Waltham, MA, USA) to remove residual genomic DNA then 200 ng of RNA was used as a template for cDNA synthesis using SuperScript IV First strand Synthesis System (Invitrogen, Carlsbad, CA, USA). Approximately 1 µg of RNA was used as a template for cDNA synthesis using a SMARTer RACE 5′/3′-kit (Clontech, Mountain View, CA, USA).

### 5′- and 3′-Rapid Amplification of cDNA Ends

To determine the TSSs of the marsupial and monotreme *DNMT3A* transcripts, 5′-RACE experiments were performed using the SMARTer RACE 5′/3′-kit (Clontech). Putative tammar and platypus *DNMT3A* sequence were searched by a blast search of the wallaby genome (Wallabase: https://wallabase.org) and the platypus genome (mOrnAna1.p.v4), respectively. After identifying the putative sequences, gene-specific primers were designed for the downstream RACE experiments. The first round RACE reaction was performed with adult tammar testis or adult platypus testis cDNA using SeqAmp DNA Polymerase (Clontech) with the gene-specific primers (supplementary table S1). The nested 5′- and 3′-RACE was performed by GoTaq DNA polymerase (Promega, Fitchburg, WI, USA) and the RACE products cloned using pGEM-T Easy Vector (Promega) and JM109 competent cells (Promega). Plasmids were extracted using Wizard Plus SV Minipreps DNA Purification System (Promega) and sequenced by Sanger Sequencing method with the vector specific primers (supplementary table S1).

### Genomic Comparison of DNMT3A Locus Across Vertebrates

Genomic sequences of several vertebrate *DNMT3As* [mouse (*Mus musculus*), human (*Homo sapiens*), tammar (*M. eugenii*), platypus (*O. anatinus*), chicken (*G. gallus*), and the bearded dragon (*P. vitticeps*)] including up to 3,000 bases of upstream sequence of the TSS was downloaded from NCBI (https://www.ncbi.nlm.nih.gov) and Wallabase (https://wallabase.org). To locate conserved genomic region across vertebrates, genomic alignments and visualization were performed by AliTV (Ankenbrand et al. 2017). To identify conserved transcription factor binding sites, the identified conserved region across mammals was subjected to multiple alignment by clustalW. Regions containing five or more highly conserved sequences around the TSS of *DNMT3A2* across mammals were identified and compared with known transcription factor binding motifs by motif searches with JASPR CORE database (2020) using TomTom function in the MEME Suite (https://meme-suite.org/meme/index.html;Bailey et al. 2015). Motifs with an *E*-value <0.05 and *q*-value <0.05 were considered as potential transcription factor binding motifs.

### Transcriptome Analysis

Publicly available raw RNA-seq data sets of koala (GEO accession: GSE128122; Yu et al. 2019), platypus (GEO accession: GSE97367; Marin et al. 2017), chicken (GEO accession: GSE97367; Marin et al. 2017), and the bearded dragon (BioProject accession: PRJEB5206; Georges et al. 2015) were downloaded from NCBI SRA (https://www.ncbi.nlm.nih.gov/sra). All RNA-seq reads were trimmed using TrimGalore! (v0.6.5; https://github.com/FelixKrueger/TrimGalore) with default settings to eliminate adaptor sequences, poor quality reads, and very short (<20 bp) reads. The trimmed reads were aligned to each genome (koala: phaCin_unsw_v4.1; platypus: mOrnAna1.p.v1; chicken: GRCg6a; the bearded dragon: pvi1.1) using HISAT2 (Kim et al. 2019). Biological replicates were merged to detect splicing variants by increasing sequence depth. The reads were used as inputs of ggsashimi program (Garrido-Martín et al. 2018) to visualize splicing junctions.

### Reverse transcription Quantitative RT-PCR

Reverse transcription quantitative RT-PCR (RT-qPCR) primers (supplementary table S1) were designed from the known tammar *DNMT3A* and *DNMT3A2* sequences and amplified 113 and 76 bp products, respectively. RT-qPCR was performed on a Quantstudio 5 (Thermo Fisher Scientific) on gonadal cDNA of both sexes from day 10 pp to adult using the SYBR Green PCR kit (QIAGEN, Hilden, Germany). TATA-box binding protein (*TBP*) and hydroxymethylbilane synthase (*HMBS*) were selected as the housekeeping genes. Expression levels of the *DNMT3As* were normalized to the geometric mean of the expression levels of housekeeping genes. Differences in the gene expression levels between stages were analyzed by Tukey–Kramer’s honestly significant difference test in R.

### Immunohistochemistry

Extensive testing of different antibody concentrations and pretreatments (enzyme digestion and heat treatment in different buffers) using various tammar tissues and organs was performed to optimize each antibody. PFA-fixed, paraffin-embedded wallaby gonads were sectioned at 6 μm and sections were mounted on Superfrost slides (Thermo Fisher Scientific, Victoria, Australia). Slides were deparaffinized, rehydrated, permeabilized in 0.1% (v/v) Triton X-100 in tris-buffered saline (TBS; 50 mM Tris HCl, 150 mM NaCl, pH 7.4) for 15 min and then blocked in 6% hydrogen peroxide in water for 10 min. Slides requiring heat treatment were heated in a water bath at 99 °C in the appropriate buffer (supplementary table S2) for 30 min and then left at room temperature for another 30 min to cool. Nonspecific staining was blocked using 10% serum from the appropriate species for 30 min at room temperature. Primary antibodies were diluted as described in supplementary table S2 in TBS/0.1% bovine-serum albumin/5% appropriate serum and then applied to the sections for 16 h at 4 °C. Negative control sections were incubated with an equivalent concentration of the appropriate immunoglobulin. Signal was amplified using biotinylated goat anti-rabbit or rabbit anti-mouse secondary antibodies and then an ABC/HRP kit (DAKO, New South Wales, Australia) and visualized with DAB (Sigma). Sections were lightly counterstained in hematoxylin and then coverslipped in DPX. At least three (usually four to five) gonads were stained for each stage for each antibody. Sections were viewed using an Olympus BX51 System microscope and photographed using an Olympus DP70 digital microscope camera.

### Western Blot

Protein was extracted from day 64 pp tammar PY testis (*n* = 4 pooled), day 80 pp ovary (*n* = 5 pooled), and day 23 of gestation fetus (*n* = 2 pooled) by homogenization in RIPA buffer (50 mM Tris base, 150 mM NaCl, 1% NP-40, 0.25% Na-deoxycholate). Fifty micrograms of each sample were electrophoresed in a 7.5% SDS-PAGE gel and then transferred to a PVDF membrane (Amersham Hybond-P). The membrane was blocked in 5% skim milk dissolved in TBST (TBS, 50 mM Tris HCl, 150 mM NaCl, pH 7.5 with 0.1% Tween 20) at 4 °C overnight and then incubated at room temperature with the anti-DNMT3A antibody (ab13888, 2 µg/ml) for 2 h, followed by a 1 h incubation with a HRP-conjugated goat anti-mouse secondary antibody diluted in TBS with 5% skim milk and 0.1% Triton X-100. Chemiluminescence using the Amersham ECL Western Blotting Detection Reagents kit (GE Healthcare Life Sciences) was used to detect bound antibody.

## Supplementary Material

Supplementary figure and supplementary table are available at *Genome Biology and Evolution* online (http://www.gbe.oxfordjournals.org/).

## Supplementary Material

evac094_Supplementary_Data

## Data Availability

Raw RNA-seq data sets of koala (GEO accession: GSE128122), platypus (GEO accession: GSE97367), chicken (GEO accession: GSE97367), and the bearded dragon (BioProject accession: PRJEB5206) were publicly available on NCBI SRA (https://www.ncbi.nlm.nih.gov/sra).

## References

[evac094-B1] Ankenbrand MJ , Hohlfeld S, Hackl T, Förster F. 2017. AliTV—interactive visualization of whole genome comparisons. Peer J Comput Sci. 3:e116.

[evac094-B2] Auclair G , Weber M. 2012. Mechanisms of DNA methylation and demethylation in mammals. Biochimie. 94:2202–2211. 22634371 10.1016/j.biochi.2012.05.016

[evac094-B3] Bailey TL , Johnson J, Grant CE, Noble WS. 2015. The MEME suite. Nucleic Acids Res. 43:W39–W49. 25953851 10.1093/nar/gkv416 PMC4489269

[evac094-B4] Bartolomei MS , Ferguson-Smith AC. 2011. Mammalian genomic imprinting. Cold Spring Harb Perspect Biol. 3:a002592. 21576252 10.1101/cshperspect.a002592 PMC3119911

[evac094-B5] Bourc’his D , Xu G-L, Lin C-S, Bollman B, Bestor TH. 2001. Dnmt3L and the establishment of maternal genomic imprints. Science (1979). 294:2536–2539. 10.1126/science.1065848 11719692

[evac094-B6] Chen T , Ueda Y, Xie S, Li E. 2002. A novel Dnmt3a isoform produced from an alternative promoter localizes to euchromatin and its expression correlates with active de novo methylation. J Biol Chem. 277:38746–38754. 12138111 10.1074/jbc.M205312200

[evac094-B7] Cúneo R , et al 2013. High-precision U-Pb geochronology and a new chronostratigraphy for the Cañadón Asfalto Basin, Chubut, central Patagonia: Implications for terrestrial faunal and floral evolution in Jurassic. Gondwana Res. 24:1267–1275.

[evac094-B8] Das R , et al 2012. Convergent and divergent evolution of genomic imprinting in the marsupial Monodelphis domestica. BMC Genomics. 13:394. 22899817 10.1186/1471-2164-13-394 PMC3507640

[evac094-B9] Delgado I , et al 2021. Control of mouse limb initiation and antero-posterior patterning by Meis transcription factors. Nat Commun. 12:1. 34035267 10.1038/s41467-021-23373-9 PMC8149412

[evac094-B10] Ferguson-Smith AC . 2011. Genomic imprinting: The emergence of an epigenetic paradigm. Nat Rev Genet. 12:565–575. 21765458 10.1038/nrg3032

[evac094-B11] Frésard L , et al 2014. Transcriptome-wide investigation of genomic imprinting in chicken. Nucleic Acids Res. 42:3768–3782. 24452801 10.1093/nar/gkt1390 PMC3973300

[evac094-B12] Frost JM , Moore GE. 2010. The importance of imprinting in the human placenta. PLoS Genet. 6:1–9. 10.1371/journal.pgen.1001015 PMC2895656 20617174

[evac094-B13] Garrido-Martín D , Palumbo E, Guigó R, Breschi A. 2018. ggsashimi: Sashimi plot revised for browser- and annotation-independent splicing visualization. PLoS Comput Biol. 14:1–6. 10.1371/journal.pcbi.1006360 PMC6114895 30118475

[evac094-B14] Georges A , et al 2015. High-coverage sequencing and annotated assembly of the genome of the Australian dragon lizard *Pogona vitticeps*. Gigascience 4:45. 26421146 10.1186/s13742-015-0085-2 PMC4585809

[evac094-B15] Glass JL , Fazzari MJ, Ferguson-Smith AC, Greally JM. 2009. CG dinucleotide periodicities recognized by the Dnmt3a-Dnmt3L complex are distinctive at retroelements and imprinted domains. Mamm Genome. 20:633–643. 19921333 10.1007/s00335-009-9232-3

[evac094-B16] Griffith OW , Brandley MC, Belov K, Thompson MB. 2016. Allelic expression of mammalian imprinted genes in a matrotrophic lizard, *Pseudemoia entrecasteauxii*. Dev Genes Evol. 226:79–85. 26943808 10.1007/s00427-016-0531-x

[evac094-B17] Hata K , Okano M, Lei H, Li E. 2002. Dnmt3L cooperates with the Dnmt3 family of de novo DNA methyltransferases to establish maternal imprints in mice. Development 129:1983–1993. 11934864 10.1242/dev.129.8.1983

[evac094-B18] Ishihara T , Griffith OW, Tarulli GA, Renfree MB. 2021. Male germline development in the tammar wallaby, *Macropus eugenii*. Reproduction 161:333–341. 33486468 10.1530/REP-20-0634

[evac094-B19] Ishihara T , Hickford D, Shaw G, Pask AJ, Renfree MB. 2019. DNA methylation dynamics in the germline of the marsupial tammar wallaby, *Macropus eugenii*. DNA Res. 26:85–94. 30535324 10.1093/dnares/dsy040 PMC6379045

[evac094-B20] Joshi RS , et al 2016. DNA methylation profiling of uniparental disomy subjects provides a map of parental epigenetic bias in the human genome. Am J Hum Genet. 99:555–566. 27569549 10.1016/j.ajhg.2016.06.032 PMC5011056

[evac094-B21] Kaneda M , et al 2004. Essential role for de novo DNA methyltransferase Dnmt3a in paternal and maternal imprinting. Nature 429:900–903. 15215868 10.1038/nature02633

[evac094-B22] Keverne EB , Curley JP. 2008. Epigenetics, brain evolution and behaviour. Front Neuroendocrinol. 29:398–412. 18439660 10.1016/j.yfrne.2008.03.001

[evac094-B23] Kim D , Paggi JM, Park C, Bennett C, Salzberg SL. 2019. Graph-based genome alignment and genotyping with HISAT2 and HISAT-genotype. Nat Biotechnol. 37:907–915. 31375807 10.1038/s41587-019-0201-4 PMC7605509

[evac094-B24] la Salle S , et al 2004. Windows for sex-specific methylation marked by DNA methyltransferase expression profiles in mouse germ cells. Dev Biol. 268:403–415. 15063176 10.1016/j.ydbio.2003.12.031

[evac094-B25] la Salle S , et al 2007. Loss of spermatogonia and wide-spread DNA methylation defects in newborn male mice deficient in DNMT3L. BMC Dev Biol. 7:104. 17875220 10.1186/1471-213X-7-104 PMC2212652

[evac094-B26] Lefebvre L , et al 1998. Abnormal maternal behaviour and growth retardation associated with loss of the imprinted gene Mest. Nat. Genet. 20:163–169. 9771709 10.1038/2464

[evac094-B27] Lefebvre L , Viville S, Barton SC, Ishino F, Surani MA. 1997. Genomic structure and parent-of-origin-specific methylation of Peg1. Hum Mol Genet. 6:1907–1915. 9302270 10.1093/hmg/6.11.1907

[evac094-B28] Li T , et al 2002. An imprinted PEG1/MEST antisense expressed predominantly in human testis and in mature spermatozoa. J Biol Chem. 277:13518–13527. 11821432 10.1074/jbc.M200458200

[evac094-B29] Li J , et al 2004. Imprinting of the human L3MBTL gene, a polycomb family member located in a region of chromosome 20 deleted in human myeloid malignancies. Proc Natl Acad Sci U S A. 101:7341–7346. 15123827 10.1073/pnas.0308195101 PMC409920

[evac094-B30] Lucifero D , et al 2007. Coordinate regulation of DNA methyltransferase expression during oogenesis. BMC Dev Biol. 7:36. 17445268 10.1186/1471-213X-7-36 PMC1878483

[evac094-B31] Lucifero D , Mann MRW, Bartolomei MS, Trasler JM. 2004. Gene-specific timing and epigenetic memory in oocyte imprinting. Hum Mol Genet. 13:839–849. 14998934 10.1093/hmg/ddh104

[evac094-B32] Ma P , de Waal E, Weaver JR, Bartolomei MS, Schultz RM. 2015. A DNMT3A2-HDAC2 complex is essential for genomic imprinting and genome integrity in mouse oocytes. Cell Rep. 13:1552–1560. 26586441 10.1016/j.celrep.2015.10.031 PMC4662907

[evac094-B33] Marin R , et al 2017. Convergent origination of a *Drosophila*-like dosage compensation mechanism in a reptile lineage. Genome Res. 27:1974–1987. 29133310 10.1101/gr.223727.117 PMC5741051

[evac094-B34] Mattiske D , Shaw G, Shaw JM. 2002. Influence of donor age on development of gonadal tissue from pouch young of the tammar wallaby, *Macropus eugenii*, after cryopreservation and xenografting into mice. Reproduction 123:143–153. 11869196 10.1530/rep.0.1230143

[evac094-B35] Monk D . 2015. Germline-derived DNA methylation and early embryo epigenetic reprogramming: The selected survival of imprints. Int J Biochem Cell Biol. 67:128–138. 25966912 10.1016/j.biocel.2015.04.014

[evac094-B36] Nimura K , et al 2006. Dnmt3a2 targets endogenous Dnmt3L to ES cell chromatin and induces regional DNA methylation. Genes Cells 11:1225–1237. 16999741 10.1111/j.1365-2443.2006.01012.x

[evac094-B37] Nishikawa K , et al 2015. DNA methyltransferase 3a regulates osteoclast differentiation by coupling to an S-adenosylmethionine-producing metabolic pathway. Nat Med. 21:281–287. 25706873 10.1038/nm.3774

[evac094-B38] Okano M , Bell DW, Haber DA, Li E. 1999. DNA methyltransferases Dnmt3a and Dnmt3b are essential for de novo methylation and mammalian development. Cell 99:247–257. 10555141 10.1016/s0092-8674(00)81656-6

[evac094-B39] Ono R , et al 2006. Deletion of Peg10, an imprinted gene acquired from a retrotransposon, causes early embryonic lethality. Nat Genet. 38:101–106. 16341224 10.1038/ng1699

[evac094-B40] Pask AJ , et al 2009. Analysis of the platypus genome suggests a transposon origin for mammalian imprinting. Genome Biol. 10:R1. 19121219 10.1186/gb-2009-10-1-r1 PMC2687786

[evac094-B41] Piedrahita JA . 2011. The role of imprinted genes in fetal growth abnormalities. Birth Defects Res A Clin Mol Teratol. 91:682–692. 21648055 10.1002/bdra.20795 PMC3189628

[evac094-B42] Plagge A , et al 2004. The imprinted signaling protein XLαs is required for postnatal adaptation to feeding. Nat Genet. 36:818–826. 15273686 10.1038/ng1397

[evac094-B43] Rada-Iglesias A , et al 2008. Whole-genome maps of USF1 and USF2 binding and histone H3 acetylation reveal new aspects of promoter structure and candidate genes for common human disorders. Genome Res. 18:380–392. 18230803 10.1101/gr.6880908 PMC2259102

[evac094-B44] Reik W , Walter J. 2001. Evolution of imprinting mechanisms: The battle of the sexes begins in the zygote. Nat Genet. 27:255–256. 11242103 10.1038/85804

[evac094-B45] Renfree MB , Ager EI, Shaw G, Pask AJ. 2008. Genomic imprinting in marsupial placentation. Reproduction 136:523–531. 18805821 10.1530/REP-08-0264

[evac094-B46] Renfree MB , Hore TA, Shaw G, Marshall Graves JA, Pask AJ. 2009. Evolution of genomic imprinting: Insights from marsupials and monotremes. Annu Rev Genomics Hum Genet. 10:241–262. 19630559 10.1146/annurev-genom-082908-150026

[evac094-B47] Riesewijk AM , Hu L, Schulz U, Tariverdian G, Ho P. 1997. Monoallelic expression of human PEG1/MEST is paralleled by parent-specific methylation in fetuses. Gene. 244:236–244. 10.1006/geno.1997.4731 9192843

[evac094-B48] Sadakierska-Chudy A , Kostrzewa RM, Filip M. 2015. A comprehensive view of the epigenetic landscape part I: DNA methylation, passive and active DNA demethylation pathways and histone variants. Neurotox Res. 27:84–97. 25362550 10.1007/s12640-014-9497-5 PMC4286137

[evac094-B49] Saitou M , Kagiwada S, Kurimoto K. 2012. Epigenetic reprogramming in mouse pre-implantation development and primordial germ cells. Development 139:15–31. 22147951 10.1242/dev.050849

[evac094-B50] Sakai Y , Suetake I, Shinozaki F, Yamashina S, Tajima S. 2004. Co-expression of de novo DNA methyltransferases Dnmt3a2 and Dnmt3L in gonocytes of mouse embryos. Gene Expr Patterns. 5:231–237. 15567719 10.1016/j.modgep.2004.07.011

[evac094-B51] Sano S , et al 2018. CRISPR-mediated gene editing to assess the roles of TET2 and DNMT3A in clonal hematopoiesis and cardiovascular disease. Circ Res. 123:335–341. 29728415 10.1161/CIRCRESAHA.118.313225 PMC6054544

[evac094-B52] Seisenberger S , et al 2012. The dynamics of genome-wide DNA methylation reprogramming in mouse primordial germ cells. Mol Cell 48:849–862. 23219530 10.1016/j.molcel.2012.11.001 PMC3533687

[evac094-B53] Singh MK , et al 2005. The T-box transcription factor Tbx15 is required for skeletal development. Mech Dev. 122:131–144. 15652702 10.1016/j.mod.2004.10.011

[evac094-B54] Smits G , et al 2008. Conservation of the H19 noncoding RNA and H19-IGF2 imprinting mechanism in therians. Nat Genet. 40:971–976. 18587395 10.1038/ng.168

[evac094-B55] Steimle JD , Moskowitz IP. 2017. TBX5: A key regulator of heart development. Curr Top Dev Biol. 122: 195–221. 28057264 10.1016/bs.ctdb.2016.08.008 PMC5371404

[evac094-B56] Stöger R , et al 1993. Maternal-specific methylation of the imprinted mouse Igf2r locus identifies the expressed locus as carrying the imprinting signal. Cell. 73:61–71. 8462104 10.1016/0092-8674(93)90160-r

[evac094-B57] Suetake I , Morimoto Y, Fuchikami T, Abe K, Tajima S. 2006. Stimulation effect of Dnmt3L on the DNA methylation activity of Dnmt3a2. J Biochem. 140:553–559. 16945937 10.1093/jb/mvj185

[evac094-B58] Suzuki S , et al 2005. Genomic imprinting of IGF2, p57KIP2 and PEG1/MEST in a marsupial, the tammar wallaby. Mech Dev. 122:213–222. 15652708 10.1016/j.mod.2004.10.003

[evac094-B59] Suzuki S , et al 2007. Retrotransposon silencing by DNA methylation can drive mammalian genomic imprinting. PLoS Genet. 3:e55. 17432937 10.1371/journal.pgen.0030055 PMC1851980

[evac094-B60] Suzuki S , Shaw G, Renfree MB. 2018. Identification of a novel antisense noncoding RNA, ALID, transcribed from the putative imprinting control region of marsupial IGF2R. Epigenetics Chromatin 11:55. 30268152 10.1186/s13072-018-0227-8 PMC6162910

[evac094-B61] Thakur A , et al 2016. Widespread recovery of methylation at gametic imprints in hypomethylated mouse stem cells following rescue with DNMT3A2. Epigenetics Chromatin 9:1–15. 27895716 10.1186/s13072-016-0104-2 PMC5118886

[evac094-B62] Ullmann SL , Shaw G, Alcorn GT, Renfree MB. 1997. Migration of primordial germ cells to the developing gonadal ridges in the tammar wallaby *Macropus eugenii*. J Reprod Fertil. 110:135–143. 9227367 10.1530/jrf.0.1100135

[evac094-B63] Watanabe K , Yokota K, Yoshida K, Matsumoto A, Iwamoto S. 2019. A novel upstream transcription factor 1 target gene N4bp2l1 that regulates adipogenesis. Biochem Biophys Rep. 20:100676. 31440585 10.1016/j.bbrep.2019.100676 PMC6698772

[evac094-B64] Weidman JR , Dolinoy DC, Maloney KA, Cheng JF, Jirtle RL. 2006. Imprinting of opossum Igf2r in the absence of differential methylation and Air. Epigenetics. 1:49–54. 17998818 10.4161/epi.1.1.2592

[evac094-B65] Yokomine T , Hata K, Tsudzuki M, Sasaki H. 2006. Evolution of the vertebrate DNMT3 gene family: A possible link between existence of DNMT3L and genomic imprinting. Cytogenet Genome Res. 113:75–80. 16575165 10.1159/000090817

[evac094-B66] Yu T , et al 2019. The piRNA response to retroviral invasion of the koala genome. Cell 179:632–643.e12. 31607510 10.1016/j.cell.2019.09.002 PMC6800666

