# Supplementary material for: Evolution of the Short Form of DNMT3A, DNMT3A2, Occurred in the Common Ancestor of Mammals
Source: Genome Biol Evol. 2022 Jun 23;14(7):evac094. doi: 10.1093/gbe/evac094 (PMC9254654; doi:10.1093/gbe/evac094)
Supplement: evac094_Supplementary_Data [file evac094_supplementary_data.docx]

**Supplementary Figure S1. DNA sequence alignment of mouse Dnmt3a/3a2 and tammar putative DNMT3A.** *M. eugenii* *DNMT3A* is derived from this study. *M. musculus* Dnmt3a (NP_001357740.1), *M. musculus* Dnmt3a2 (NP_001297053.1), were obtained from the NCBI data base. Green colours represent highly conserved exons between mouse *Dnmt3a/3a2* and the tammar *DNMT3A*.

**Supplementary Figure S2. Western blotting analysis of the anti-Human DNMT3A/DNMT3A2 in the tammar.** Western blotting was performed using tissue lysates derived from day 64 pp tammar PY testis (n=4 pooled), day 80 pp ovary (n=5 pooled), and day 23 of gestation fetus (n=2 pooled). The antibody used in this study could detect both DNMT3A and DNMT3A2.

**Supplementary TableS1. Primers used for this study**

| Primer | Sequence |
| --- | --- |
| 5'/3' Race reaction |  |
| *DNMT3A* (Tammar) |  |
| 5'RACE | 5'-TACTGATAGGCACACTCCAGGAAGC-3' |
| 5'RACE nested | 5'-CAGTAGGTTGCTGTAAAGCAGG-3' |
| 3'RACE | 5'-AGAGCACAGCAGAAAAGCCCAAGAT-3' |
| 3'RACE nested | 5'-ACGGCTCTTCTTTGAGTTCTACCGC-3' |
| *DNMT3A* (Platypus) |  |
| 5'RACE | 5'-CCCATCTCCGAACCACATAACCCAG-3' |
| 5'RACE nested | 5'-TTCCTGTACATGGGCTGCTTGTTGT-3' |
| 3'RACE | 5'-GCTGATGCCTTTGAGTTCGTTCTGC-3' |
| 3'RACE nested | 5'- GGTGGACCGTTACATTGCCTCAGAA-3' |
| RT-PCR |  |
| *DNMT3A* (Tammar) |  |
| Forward | 5'-AAAACAGCGACACCCCTAAG-3' |
| Reverse | 5'-CTTCAGCACCAATAGTCCTGTGGCA-3' |
| *DNMT3A2* (Tammar) |  |
| Forward | 5'-CACAGTCTGAGCTTCTGGGCCAAG-3' |
| Reverse | 5'-CTTCAGCACCAATAGTCCTGTGGCA-3' |
| *DNMT3A2* (Platypus) |  |
| Forward | 5'-ATCTCCCCCTTTCCCCTCCCCCAAG-3' |
| Reverse | 5'-TGCAGAACGAACTCAGAGGCATCAG-3' |
| *DNMT3AOa* (Platypus) |  |
| Forward | 5'-CACCCCTCTCCAGGACTGAGATCTC-3' |
| Reverse | 5'-CACTCTCGTCATTTTCAGGGCAGGT-3' |
| RT-qPCR |  |
| *DNMT3A* (Tammar) |  |
| Forward | 5'-AAAACAGCGACACCCCTAAG-3' |
| Reverse | 5'-TTCACTGAGCTTCTCCACATCC-3' |
| *DNMT3A2* (Tammar) |  |
| Forward | 5'-TTGCACCTGGCCTTATGGG-3' |
| Reverse | 5'-TCCCCTGGGTTTCTTCTACAAC-3' |
| *HMBS* |  |
| Forward | 5'-﻿ACCTGACTGGAGGAGTATGGAGT-3' |
| Reverse | 5'-﻿TGGGCTAAGATGTTGACGGTTGT-3' |
| *TBP* |  |
| Forward | 5'-﻿GGACAAACTGAAGCAAAGGGACC-3' |
| Reverse | 5'-﻿AGGGCATCATTGGGCTAAAGATAG-3' |

**Supplementary Table S2. Antibody used in this study**

| Type | Source | Treatment | Working concentration | Description of epitope |
| --- | --- | --- | --- | --- |
| Mouse monoclonal (Clone 64B1446) | Abcam  (Ab13888) | 1 mM EDTA (pH 8) | (6.6 ug/ml) | AA 705-908 of mouse Dnmt3a |
